# Gonadal androgens are associated with decreased type I interferon production by plasmacytoid dendritic cells and increased IgG titres to BNT162b2 following co-vaccination with live attenuated influenza vaccine in adolescents

**DOI:** 10.3389/fimmu.2024.1329805

**Published:** 2024-02-28

**Authors:** Oliver L. Sampson, Cecilia Jay, Emily Adland, Anna Csala, Nicholas Lim, Stella M. Ebbrecht, Lorna C. Gilligan, Angela E. Taylor, Sherley Sherafin George, Stephanie Longet, Lucy C. Jones, Ellie Barnes, John Frater, Paul Klenerman, Susie Dunachie, Miles Carrol, James Hawley, Wiebke Arlt, Andreas Groll, Philip Goulder

**Affiliations:** ^1^ Peter Medawar Building for Pathogen Research, University of Oxford, Oxford, United Kingdom; ^2^ Department of Statistics, Technical University of Dortmund, Dortmund, Germany; ^3^ Steroid Metabolome Analysis Core, Institute of Metabolism and Systems Research, University of Birmingham, Birmingham, United Kingdom; ^4^ Biochemistry Department, Clinical Science Building, Wythenshawe Hospital, Manchester, United Kingdom; ^5^ Wellcome Trust Centre for Human Genetics, University of Oxford, Oxford, United Kingdom; ^6^ Department of Microbiology, Division of Infection and Immunity, Cardiff University, Cardiff, United Kingdom; ^7^ Medical Research Council London Institute of Medical Sciences (MRC LMS), Imperial College London, London, United Kingdom

**Keywords:** type I interferon, plasmacytoid dendritic cell, immune sex difference, androgen, adolescent vaccination

## Abstract

mRNA vaccine technologies introduced following the SARS-CoV-2 pandemic have highlighted the need to better understand the interaction of adjuvants and the early innate immune response. Type I interferon (IFN-I) is an integral part of this early innate response that primes several components of the adaptive immune response. Women are widely reported to respond better than men to tri- and quadrivalent influenza vaccines. Plasmacytoid dendritic cells (pDCs) are the primary cell type responsible for IFN-I production, and female pDCs produce more IFN-I than male pDCs since the upstream pattern recognition receptor Toll-like receptor 7 (TLR7) is encoded by X chromosome and is biallelically expressed by up to 30% of female immune cells. Additionally, the TLR7 promoter contains several putative androgen response elements, and androgens have been reported to suppress pDC IFN-I *in vitro*. Unexpectedly, therefore, we recently observed that male adolescents mount stronger antibody responses to the Pfizer BNT162b2 mRNA vaccine than female adolescents after controlling for natural SARS-CoV-2 infection. We here examined pDC behaviour in this same cohort to determine the impact of IFN-I on anti-spike and anti-receptor-binding domain IgG titres to BNT162b2. Through flow cytometry and least absolute shrinkage and selection operator (LASSO) modelling, we determined that serum-free testosterone was associated with reduced pDC IFN-I, but contrary to the well-described immunosuppressive role for androgens, the most bioactive androgen dihydrotestosterone was associated with increased IgG titres to BNT162b2. Also unexpectedly, we observed that co-vaccination with live attenuated influenza vaccine boosted the magnitude of IgG responses to BNT162b2. Together, these data support a model where systemic IFN-I increases vaccine-mediated immune responses, yet for vaccines with intracellular stages, modulation of the local IFN-I response may alter antigen longevity and consequently improve vaccine-driven immunity.

## Introduction

The emergence of mRNA vaccine technologies in response to the SARS-CoV-2 pandemic has exposed the gap in understanding the complex interaction of vaccine adjuvants and the innate immune response in shaping the adaptive immune response and outcome of vaccination. Historic understanding of vaccine immunology is based largely on protein-subunit vaccines where empirically proven adjuvants are proposed to activate antigen-presenting cells to present vaccine antigen to CD4+ T cells, CD8+ T cells, and B cells for a robust adaptive response (1, 2). Type I interferon (IFN-I) is a family of innate antiviral proteins with powerful immunostimulatory effects, which include promoting the development of CD4+ and CD8+ T cells to effector and memory phenotypes and aiding B-cell survival and differentiation (3).

Accordingly, IFN-I is implicated in vaccine outcome, and a high initial IFN-I response correlates with increased antibody titres for both seasonal trivalent influenza vaccine (TIV) and live attenuated influenza vaccine (LAIV) in adults (4, 5) and crucially also in children (6, 7).

Further, a stronger antibody response to seasonal TIV is consistent in women despite a new formulation annually (8–12). Mechanistically, women produce greater quantities of IFN-I since the upstream receptor, Toll-like receptor 7 (TLR7), is encoded by the X chromosome and escapes X chromosome inactivation in up to 30% of female immune cells (13). The primary route of IFN-I induction by TIV is *via* TLR7, and vaccine-driven IFN-I was shown to induce vaccine-specific CD4+ T cells and B cells, which were essential for protection from lethal influenza challenge in mice (14).

Plasmacytoid dendritic cells (pDCs) are the primary source of IFN-I, and pDCs from women consistently produce greater quantities of IFN-I in response to TLR7 stimulation when compared to male pDCs (15–22).

Additionally, male androgens are broadly considered to suppress the immune response (11) and a systems analysis of seasonal TIV responses identified a negative relationship between TIV antibody titres and serum testosterone levels in adult men (10, 12, 23). Indeed, putative androgen response elements are located within the promoters of both TLR7 and the downstream myeloid differentiation primary response 88 (MyD88) signalling intermediate (12, 24), providing the potential mechanism for androgen-mediated immune suppression of pDC IFN-I.

Accordingly, female pDCs treated with dihydrotestosterone (DHT), the more bioactive product of testosterone, show reduced IFN-I production following TLR7 stimulation (25). Further, pDCs from male infants during the so-called “mini-puberty” of early infancy—when androgen levels transiently rise to pubertal levels—showed reduced IFN-I production in response to TLR7 stimulation when compared to longitudinal samples (25).

The immune system of children differs markedly from that of adults, and healthy children have higher IFN-I responses than adults, including to natural SARS-CoV-2 infection, which has been linked to their increased capacity to clear the virus (26). Additionally, a stronger early IFN-I response was predictive of increased neutralising antibody titres following Oxford/AstraZeneca ChAdOx-nCoV19 (AZD1222) vaccination (27).

In autumn 2021, adolescent children in the UK were offered a primary dose of the Pfizer BNT162b2 mRNA SARS-CoV-2 vaccine alongside optional co-vaccination with live attenuated influenza vaccine as part of the national governmental pandemic response. A key immunological feature of BNT162b2 is a strong early IFN-I response (28), but no difference in the outcome of vaccination between sexes has been reported in studies of adults (29–31) with no such data available for children. Likewise, LAIV has been approved for use in children in the UK since 2013, but we are aware of no study to date reporting differential outcomes between sexes in adults or children.

We recently published a study of a cohort of adolescents receiving their primary dose of Pfizer BNT162b2 in autumn 2021 as part of the national programme. Contrary to that expected considering seasonal influenza responses, anti-spike and anti-receptor-binding domain (anti-RBD) IgG titres were stronger in men than women (32).

In the present study, we examined the pDC response following vaccination in 33 of these adolescents (aged 12 years to 16 years) to investigate whether sex differential IFN-I production drives a differential outcome to vaccination in adolescents. pDCs were chosen, as they have a readily observable sex difference in their primary phenotype, IFN-I production, which is directly linked to their response to exogenous RNA, as introduced by BNT162b2 vaccination.

## Results

### Cohort demographics

In autumn 2021, adolescent school children in the UK were offered a primary dose of Pfizer BNT162b2 alongside optional co-administration of intranasal LAIV. Children from three schools in Oxfordshire were enrolled in a longitudinal study of the vaccine response, which has been published elsewhere (32). Additional blood samples were taken from 33 adolescents on the day of vaccination (“V1”), and the baseline phenotype of their pDCs was measured *via* a whole blood TLR7 stimulation assay (22). Further samples were obtained from 18/33 of these adolescents the day following vaccination, and the whole blood assay was repeated to determine the influence of vaccination on the early IFN-I response by pDCs. Of the 33 adolescents, all were vaccinated with BNT162b2, and 25 were also co-vaccinated with LAIV. Cohort demographics are outlined in **Table 1**.

**Table 1:** Cohort demographics.

	Male	Female	Total
**Participants**	18 (7)	15 (11)	33 (18)
**Age**	14 years 5 months; 12 years 2 months to 16 years 0 months	13 years 0 months; 12 years 0 months to 15 years 11 months	14 years 3 months; 12 years 0 months to 16 years 0 months
**LAIV co-vaccination**	14 (5)	11 (8)	25 (13)
**Natural SARS-CoV-2 pre-V1**	11 (2)	6 (4)	17 (6)
**Natural SARS-CoV-2 by post-V1**	12 (2)	8 (6)	20 (8)

Numbers in parentheses indicate numbers for the subset of participants who were re-sampled the day following vaccination (n = 18 out of 33). Age given as median with range.

LAIV, live attenuated influenza vaccine.

### High pDC activation and cytokine secretion following co-/vaccination

**Figure 1:**
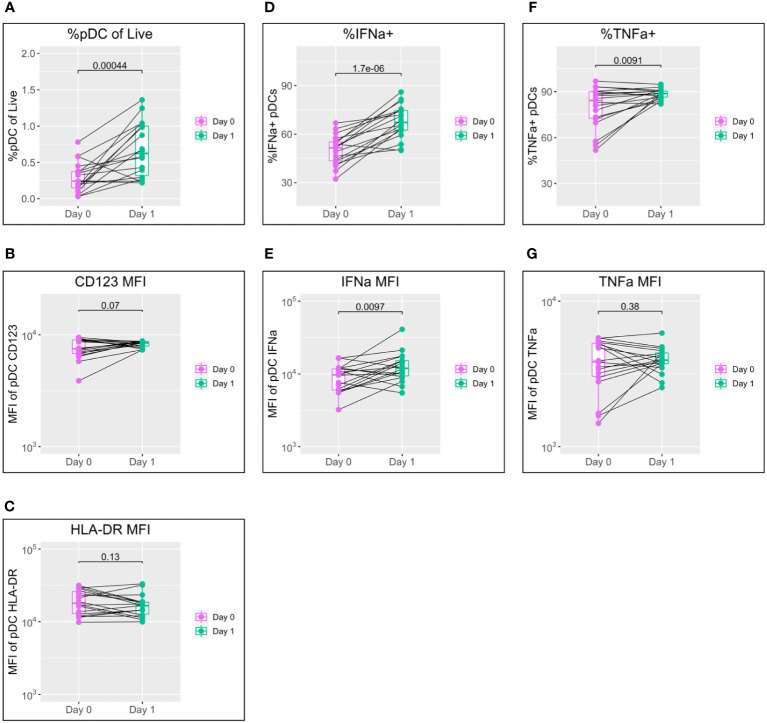
pDC activation following BNT162b2 +/− LAIV co-vaccination. pDC flow cytometry data following whole blood TLR7 stimulation for the day of vaccination (D0) and the following day (D1) (n = 18; male = 7, female = 11. **(A)** Percentage of pDCs of live PBMCs. **(B)** MFI of pDC CD123. **(C)** MFI of pDC HLA-DR. **(D)** Percentage of IFN-α+ pDCs. **(E)** MFI of pDC IFN-α. **(F)** percentage of pDC TNF-α. **(G)** MFI of pDC TNF-α. Pairwise comparisons *via* paired Student’s t-test. pDC, plasmacytoid dendritic cell; LAIV, live attenuated influenza vaccine; PBMCs, peripheral blood mononuclear cells; MFI, mean fluorescence intensity.

Since IFN-I plays a central role in initiating both the innate and adaptive immune responses, we first studied the behaviour of pDCs, as the main IFN-I-producing cell type, to TLR7 in the 18 participants sampled on both the day of vaccination and the following day (**Figure 1**; see pDC gating strategy in **Supplementary Figure S1**). This single timepoint was selected, as our previous work has shown pDC activation peaks within 24 hours of stimulation (22).

A striking increase in pDC abundance was observed between the day of vaccination (D0) and the following day (D1), with the population as a percentage of live peripheral blood mononuclear cells (PBMCs) increasing from a median of 0.24% to 0.62% (p = 0.00044) (**Figure 1A**). Increased pDC activation between D0 and D1 was also seen in a modest increase in CD123 expression but was not reflected in HLA-DR (**Figures 1B, C**).

IFN-α is the principal subtype of IFN-I, and its production by pDCs increased significantly between D0 and D1 in both the percentage of pDCs producing IFN-α (from a median of 51.5% to 67.3%; p < 0.0001) and production by individual cells measured *via* mean fluorescence intensity (MFI) (median of 9,694 to 11,937; p = 0.0097) (**Figures 1D, E**).

Production of TNF-α showed a mixed pattern in terms of the percentage of TNF-α+ pDCs and TNF-α MFI; levels tended to increase for individuals with lower D0 expression and to decrease for those with higher D0 expression (**Figures 1F, G**).

To control for variation in cytometer performance between D0 and D1 and determine genuine phenotype from assay noise, two unvaccinated control individuals were also sampled, stimulated, and stained alongside vaccinated samples on D0 and D1. pDC flow cytometry data for these samples showed only minor variation between D0 and D1 for all of pDC abundance, MFI for CD123 and HLA-DR, percentage positive for IFN-α and TNF-α, and MFI for IFN-α and TNF-α (**Supplementary Figure S2**).

### Delayed activation kinetics and IFN-I production by male pDCs

**Figure 2:**
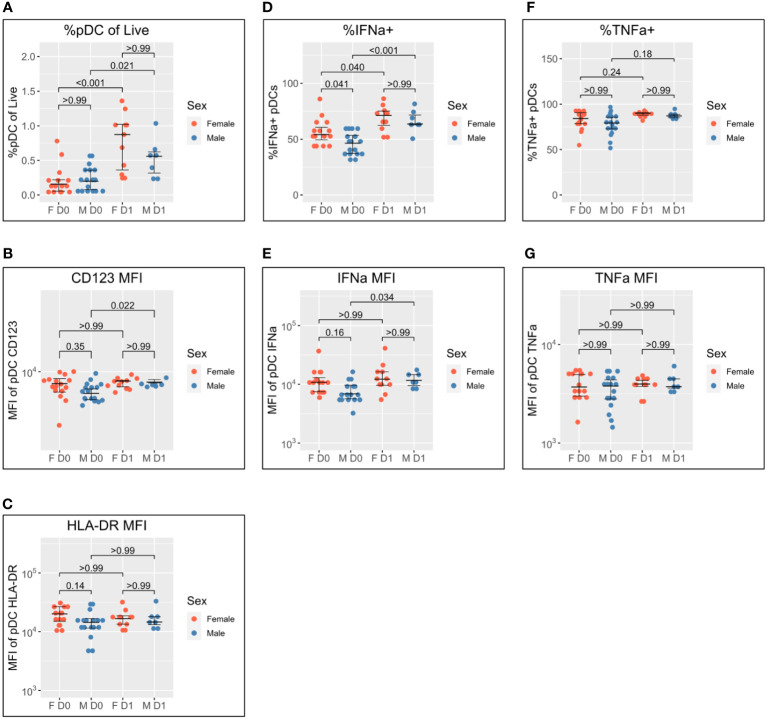
Sex differential pDC activation following BNT162b2 +/− LAIV co-vaccination. Sex-stratified pDC flow cytometry data following whole blood TLR7 stimulation for the day of vaccination (D0) (n = 33; male = 18, female = 15) and the following day (D1) (n = 18; male = 7, female = 11). **(A)** Percentage of pDCs of live PBMCs. **(B)** MFI of pDC CD123. **(C)** MFI of pDC HLA-DR. **(D)** Percentage of IFN-α+ pDCs. **(E)** MFI of pDC IFN-α. **(F)** Percentage of pDC TNF-α. **(G)** MFI of pDC TNF-α. Pairwise comparisons *via* unpaired Student’s t-test with Bonferroni’s correction for multiple comparisons. pDC, plasmacytoid dendritic cell; LAIV, live attenuated influenza vaccine; PBMCs, peripheral blood mononuclear cells; MFI, mean fluorescence intensity.

As detailed in the Introduction, female pDCs produce greater quantities of IFN-I in response to TLR7 stimulation than male pDCs, and we have recently reported that this may be underpinned by differential activation kinetics (22). We, therefore, stratified the data by sex to investigate whether differential activation kinetics were apparent between male and female pDCs following co-/vaccination in this cohort of adolescents (**Figure 2**).

pDC abundance is comparable between men and women on D0 and increases in both sexes to D1, but to a greater extent in women (median 0.16% to 0.88%; p < 0.001) than in men (median of 0.20% to 0.56%; p = 0.021) (**Figure 2A**).

pDC activation *via* CD123 appears lower on D0 for men than women but does not reach statistical significance (p = 0.35). There is a significant increase in CD123 expression between D0 and D1 for male pDCs (5204 to 6469; p = 0.022), whereas female CD123 remains consistent such that no difference is apparent between the sexes on D1 (**Figure 2B**). A similar pattern was also observed for HLA-DR where expression appeared higher for female pDCs on D0 but was comparable to that for male pDCs on D1 (**Figure 2C**).

The widely reported significantly increased IFN-α production by female pDCs is also seen here on D0 (median 54.1% compared to 46.3% for men; p = 0.041) (**Figure 1D**). The percentage of IFN-α+ pDCs increases for both sexes between D0 and D1, but there is a marginally greater fold-change for male pDCs [46.3% to 63.6% (1.4-fold); p < 0.001] than female pDCs [54.1% to 71.2% (1.3-fold); p = 0.040] such that there is no statistical sex difference on D1 (**Figure 2D**). The same pattern is seen for IFN-α MFI (**Figure 2E**).

Conversely, no sex difference is seen for the percentage of TNF-α+ pDCs nor TNF-α MFI either before or after co-/vaccination (**Figures 2F, G**).

### Gonadal androgens but not adrenal androgens differ between men and women and correlate with age during adolescence

**Figure 3:**
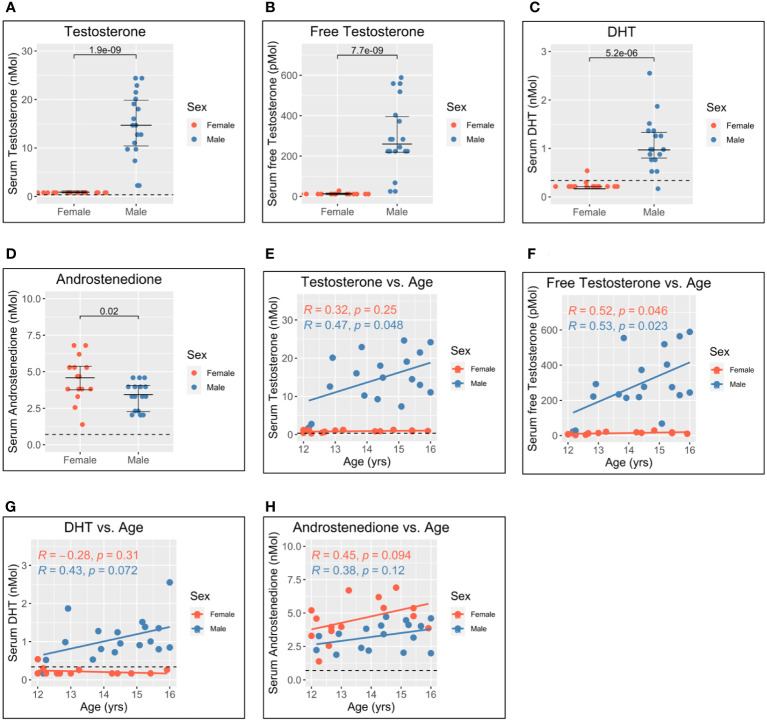
Serum gonadal androgen measurements in adolescent men and women. Serum androgen concentrations determined *via* tandem mass spectrometry for adolescents (n = 33; male = 18, female = 15). **(A–D)** Comparison of serum concentrations in men and women for testosterone, free testosterone, dihydrotestosterone (DHT), and androstenedione, respectively. **(E–H)** Correlation of androgen concentration with age for men and women for testosterone, free testosterone, DHT, and androstenedione, respectively. Pairwise comparisons *via* Student’s unpaired t-test. Correlations *via* Pearson’s method.

The pubertal age range of the adolescents studied offered a unique opportunity to investigate further the relationship between androgens and pDC IFN-I production. In addition to canonical gonadal androgen synthesis—which produces testosterone and DHT *via* the precursor androstenedione—adrenal synthesis of 11-oxygenated androgens is reported to contribute significantly to the circulating androgen pool. Serum levels of both gonadal and adrenal androgens were, therefore, determined for each participant *via* liquid chromatography–tandem mass spectrometry (**Figure 3**). Since nearly all circulating testosterone is bound by either sex hormone-binding globulin (SHBG) or albumin and is biologically unavailable to cells in the blood (33), the level of unbound, or “free”, testosterone was also calculated using measurements of SHBG and albumin (34).

As expected, levels of testosterone, free testosterone, and DHT were all significantly higher for male adolescents than female adolescents (**Figures 3A–C**), and all increased with age in men (**Figures 3E–G**). Conversely, levels of androstenedione were significantly higher for women (**Figure 3D**) and weakly correlated with age in women but not men (**Figure 3H**).

**Figure 4:**
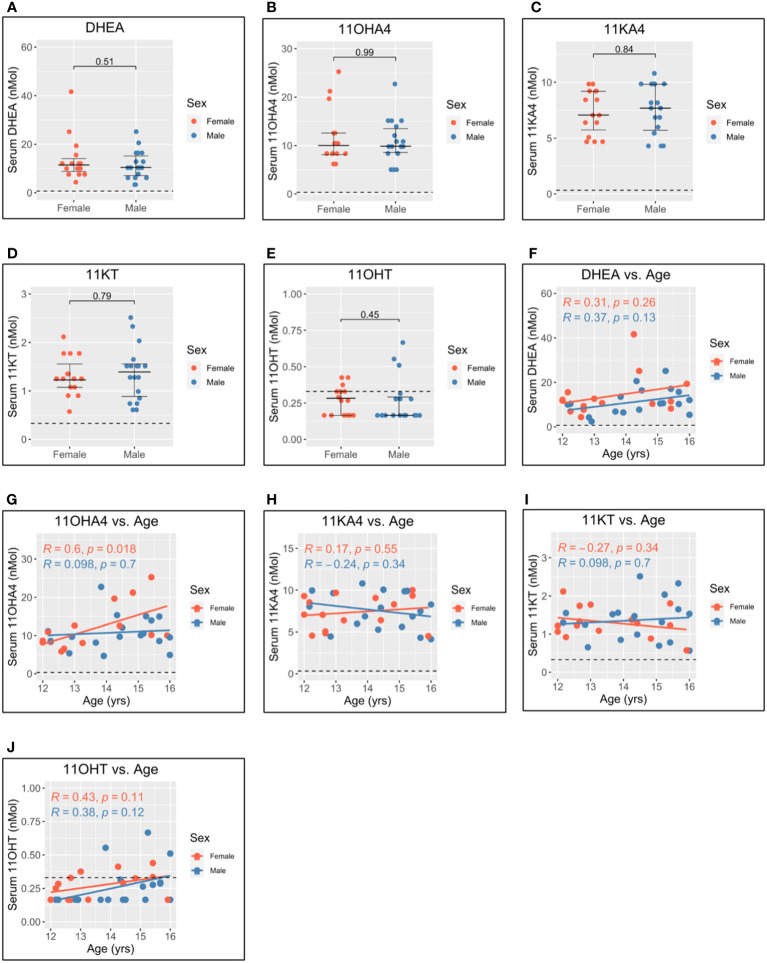
Serum adrenal androgen measurements in adolescent men and women. Serum androgen concentrations determined *via* tandem mass spectrometry for adolescents (n = 33; male = 18, female = 15). **(A–E)** Comparison of serum concentrations in men and women for dehydroepiandrosterone (DHEA), 11-hydroxyandrostenedione (11OHA4), 11-ketoandrostenedione (11KA4), 11-ketotestosterone (11KT), and 11-hydroxytestosterone (11OHT), respectively. **(F–J)** Correlation of androgen concentration with age for men and women for DHEA, 11OHA4, 11KA4, 11KT, and 11OHT, respectively. Pairwise comparisons *via* Student’s unpaired t-test. Correlations *via* Pearson’s method.

Levels of dehydroepiandrosterone (DHEA) (**Figure 4A**), 11-hydroxyandrostenedione (11OHA4) (**Figure 4B**), 11-ketoandrostenedione (11KA4) (**Figure 4C**), 11-ketotestosterone (11KT) (**Figure 4D**), and 11-hydroxytestosterone (11OHT) (**Figure 4E**) were not significantly different between men and women. This suggests that 11-oxygenated androgens and DHEA do not significantly contribute to sex differences in androgen levels. Further, 11OHA4 was the only 11-oxygenated androgen to correlate with age and increased with age in women (**Figure 4G**). DHEA (**Figure 4F**), 11KA4 (**Figure 4H**), 11KT (**Figure 4I**), and 11OHT (**Figure 4J**) did not correlate with age in this adolescent cohort.

### pDC IFN-I production is negatively associated with free testosterone

Since androgens have been implicated in a dampened vaccine response (10) and reduced IFN-I production in male infants (25), we next sought to investigate whether the androgen levels measured here are related to pDC IFN-I production. Using D0 flow cytometry data to control for any effect of co-/vaccination, a least absolute shrinkage and selection operator (LASSO) regression model (35, 36) was constructed using androgen concentrations as input co-variates and the percentage of IFN-α+ pDCs as the dependent variable to determine if a relationship exists between androgens and pDC IFN-α+ (**Table 2**).

**Table 2:** LASSO regression coefficient estimates for the effect of androgens on pDC IFN-I.

Covariate	Androgen group	Coefficient	Interpretation
Androstenedione	Gonadal	0.00	No effect
Testosterone	Gonadal	0.00	No effect
Free testosterone	Gonadal	−0.016	−1.6% %IFN-α+ pDC per 100 pmol
Dihydrotestosterone (DHT)	Gonadal	0.00	No effect
Dehydroepiandrosterone (DHEA)	Adrenal	0.00	No effect
11β-Hydroxyandrostenedione (11OHA4)	Adrenal	0.00	No effect
11-Ketoandrostenedione (11KA4)	Adrenal	0.00	No effect
11-Ketotestosterone (11KT)	Adrenal	0.00	No effect
11β-Hydroxytestosterone (11OHT)	Adrenal	0.00	No effect

LASSO, least absolute shrinkage and selection operator; pDC, plasmacytoid dendritic cell; IFN-I, type I interferon.

Only free testosterone is identified by the model as influential of pDC IFN-α and causes a reduction of 1.6% IFN-α+ pDCs per 100 pmol (**Table 2**). Moreover, this effect overshadowed a negative influence of total testosterone, which was only identified if free testosterone was excluded from the model (**Supplementary Table S1**).

### BNT162b2 IgG response is increased by LAIV co-vaccination and DHT but decreased by pDC abundance

Given the potential of IFN-I to influence each stage of the immune response to vaccination, we next turned to the relationship between the early pDC IFN-I response and the outcome of BNT162b2 vaccination. Having observed differential activation between male and female pDCs (**Figure 2**) and having identified a role for free testosterone in decreased pDC IFN-I (**Table 2**), we analysed the D0 flow cytometry data together with gonadal androgen measurements in LASSO models for post-vaccination anti-spike-IgG and anti-RBD-IgG responses measured a median of 37 days post-vaccination (**Table 3**). These IgG titres are published elsewhere (32) and were significantly higher in men than women for SARS-CoV-2-naïve adolescents, although they did not differ between the sexes for infected adolescents. Likewise, there was no sex difference in the IgG titres of the four LAIV haemagglutinin antigens (32).

**Table 3:** LASSO regression coefficient estimates for post-V1 anti-spike and anti-RBD IgG.

Covariate	Spike coefficient	Interpretation	RBD coefficient	Interpretation
Female sex	0.00	No effect	0.00	No effect
Age	0.00	No effect	0.00	No effect
LAIV co-vaccination	221,683	+2.2 × 10^5^ AU if co-vaccinated	91,350	+9.1 × 10^4^ AU if co-vaccinated
%pDC/Live	−83,379	−8.3 × 10^3^ AU per extra 0.1% pDC	−26,624	−2.6 × 10^3^ AU per extra 0.1% pDC
CD123 MFI	0.00	No effect	0.00	No effect
HLA-DR MFI	0.00	No effect	0.00	No effect
%TNF-α+	0.00	No effect	0.00	No effect
%IFN-α+	0.00	No effect	0.00	No effect
Androstenedione	0.00	No effect	0.00	No effect
Testosterone	0.00	No effect	0.00	No effect
Free testosterone	0.00	No effect	0.00	No effect
DHT	65,047	+6.5 × 10^4^ AU per nmol DHT	90,845	+9.1 × 10^4^ AU per nmol DHT

LASSO, least absolute shrinkage and selection operator; anti-RBD, anti-receptor-binding domain; LAIV, live attenuated influenza vaccine; pDC, plasmacytoid dendritic cell; MFI, mean fluorescence intensity; DHT, dihydrotestosterone.

In order to control for the additional antigen dose of natural SARS-CoV-2 infection on post-vaccination antibody titres, the cohort was separated according to evidence of natural SARS-CoV-2 infection. LASSO modelling was only possible for naturally infected adolescents (n = 20), as the small number of naïve adolescents (n = 13) approached the number of covariates and caused the model to fail.

Surprisingly, in the LASSO models for both anti-spike-IgG and anti-RBD-IgG for SARS-CoV-2-infected adolescents, the largest effect is seen for LAIV co-vaccination, which is associated with increased titres for both anti-spike-IgG and anti-RBD-IgG (**Table 3**). In contrast to reports of androgens suppressing vaccine responses, DHT is also identified as positively influencing IgG titres for both antigens. The only other association identified by the model is a negative relationship between pDC abundance and the IgG titres of both antigens (**Table 3**).

The size of the model (n = 20) precluded the inclusion of adrenal androgens as covariates, but these were found not to influence IgG titres to either antigen in separate LASSO analyses containing only gonadal and adrenal androgens (**Supplementary Table S2**).

## Discussion

This study sought to characterise the behaviour of pDCs from adolescents in response to BNT162b2 +/− LAIV co-vaccination and investigate whether a role can be attributed to differential pDC IFN-I in the widely reported sex differential outcome to vaccination, particularly to seasonal TIV (5, 7–11, 37).

pDCs were selected as the focus of this study due to their readily observable phenotypic sex difference in the production of IFN-I, which is known to be elicited by BNT162b2 vaccination (28). pDCs also produce 200- to 1,000-fold more IFN-I than any other immune cell (38) and are responsible for the majority of systemic IFN-I following respiratory infection (39), which can reach up to 95% of circulating IFN-I (40). Thus, the contribution of additional innate immune cells, such as macrophages (39), to the IFN-I response following BNT162b2 +/− LAIV co-vaccination was not considered in this study.

After BNT162b2 +/− LAIV co-vaccination in adolescents, a substantial increase in pDC abundance was observed by the following day, as was a modest increase in cell activation measured *via* CD123 expression. This accompanied a larger increase in IFN-I production, as IFN-α was significantly increased the day following vaccination.

Importantly, differences were seen in the behaviour of male and female pDCs. On the day of vaccination, male pDCs produced significantly less IFN-α than female pDCs in line with previous studies in children and adults (15–22). Male pDC IFN-α increased to a greater extent than for female pDCs the day following vaccination and was accompanied by an increase in cell activation by greater CD123 expression, whilst the abundance of pDCs increased more so for women than men. This aligns with our previous observations that biallelic TLR7 expression may allow female pDCs to more readily produce IFN-I, whilst male pDCs require a greater level of activation in order to catch up (22). Indeed, higher levels of the T-cell co-stimulatory molecule CD86 have been reported on female pDCs compared to male pDCs 20 hours following TLR7 stimulation (20). Such need for increased activation by male cells may provide one explanation for the greatest risk group for adverse effects following SARS-CoV-2 mRNA vaccination, being young men (41).

In addition, the pubertal age range of the cohort gave the unique opportunity to investigate whether the immunosuppressive properties of androgens extend to pDC IFN-I in adolescents. Men had significantly higher levels of the classic gonadal androgens, but despite reports of their contribution to the circulating androgen pool, 11-oxygenated adrenal androgens were found not to differ significantly between men and women. Indeed, LASSO modelling using the hormone data identified only free testosterone as negatively influential of pDC IFN-α. That this effect overshadowed the effect of total testosterone implies a direct antagonistic relationship between testosterone and pDC IFN-α, as nearly all circulating testosterone is protein-bound (33) and, therefore, unavailable to act on cells within the blood.

IFN-I is important for priming an adaptive immune response by activating CD4+ T-helper cells and B cells (3), and the female adaptive immune system is predominantly TH2-biased (11). It might, therefore, be anticipated from our data showing increased female pDC IFN-I following BNT162b2 +/− LAIV co-vaccination that anti-spike-IgG and anti-RBD-IgG antibody titres are also increased in women. Conversely, titres for both are higher in men for SARS-CoV-2-naïve adolescents and equal for men and women after SARS-CoV-2 infection (32). Unsurprisingly, then, we did not identify sex or any measure of pDC activation and cytokine production, including free testosterone, in LASSO models for anti-spike-IgG and anti-RBD-IgG using data from SARS-CoV-2-infected adolescents. Instead, LAIV co-vaccination was identified as the strongest positive influence of antibody titres. Somewhat more surprisingly, DHT was also identified as positively influencing antibody titres to both antigens.

A similar difference was also seen by Sparks et al. (42), who reported increased antibody titres to the quadrivalent seasonal influenza vaccine in men compared to women following natural SARS-CoV-2 infection and linked this to an increase in “virtual memory” T cells in men. Such non-specific activation of T cells unrelated to the present vaccine immune response, so-called “bystander activation”, has also been reported for Modified Vaccinia virus Ankara (MVA) (43) and Bacillus Calmette–Guerin (BCG) (44) and has been attributed to IFN-I induced by vaccines (45). LAIV is a potent inducer of IFN-I (46), and IFN-I signatures 7 days post-vaccination have previously been shown to be predictive of neutralising antibody titres to LAIV (6). Importantly, this was attributable in children to increased numbers of total naïve, memory, and transitional B cells following LAIV vaccination when compared to seasonal TIV (6), which is a weaker inducer of IFN-I relative to LAIV (46). Furthermore, LAIV was shown to offer greater protection than TIV from influenza in a paediatric cohort (47), which has been linked to a broader and longer-lasting adaptive immune response indicative of more robust systemic immune activation (48).

It follows that the boosting effect of LAIV seen here may be attributable to increased levels of systemic IFN-I leading to non-specific bystander activation of SARS-CoV-2 memory cells. That IFN-I is not directly identified in the LASSO models for anti-spike-IgG or anti-RBD-IgG may be explained by the fact that LAIV IFN-I production does not peak until 7 days post-vaccination, whereas the pDC data used in the LASSO models were from the day of vaccination.

The IFN-I elicited by LAIV can be attributed to TLR7 signalling (14, 46), and whilst IFN-I is a powerful vaccine adjuvant, excessive induction leads to hyperinflammation and autoimmunity (49). Accordingly, mRNA vaccines like BNT162b2 are formulated to minimise TLR7 signalling *via* highly efficient purification through high-pressure liquid chromatography and substitution for the hypo-agonistic synthetic nucleotide 1-methylpseudouridine (50–52). mRNA preparations with such modifications display an extended cytoplasmic half-life and translational potential *in vitro* (53–55). It follows that TLR7 activation may, therefore, increase the rate of clearance of mRNA within cells and decrease the cytoplasmic half-life of mRNA vaccines *in vivo*. Although present in circulation, DHT is mainly synthesised locally by specialised target tissues including the skin (56), and its identification as positively influential of BNT162b2 antibody titres suggests that DHT synthesised locally at the site of vaccination could reduce TLR7 signalling within vaccine-containing muscle cells. In turn, this would increase the duration of antigen production for presentation to the adaptive immune response leading to a higher titre humoral immune response.

This deviates from the current understanding of the immune-suppressive role of androgens in reducing vaccine responses in men compared to women (10, 12, 23). However, BNT162b2 antibody titres were higher in men than women for both antigens in SARS-CoV-2-naïve adolescents and were comparable for men and women after natural SARS-CoV-2 infection in this cohort (32). Likewise, no sex difference was seen for LAIV (32) and has not been reported to date in other studies of LAIV. LAIV replicates within cells of the upper airway mucosa (57), and BNT162b2 is translated within muscle cells (48). Together, these data suggest that the influence of androgens on vaccines with intracellular stages is different from the immunosuppressive role reported for seasonal protein-subunit influenza vaccines. Local modulation of the IFN-I response by androgens may, therefore, be beneficial for the outcome of such vaccines.

The main limitation of this study is that the number of adolescents studied was lower than planned due to reduced recruitment resulting from contact isolation during the SARS-CoV-2 Delta wave in autumn 2021 (58). This precluded stratification of the phenotypic data by LAIV co-vaccination and natural SARS-CoV-2 infection status, limited LASSO analyses in the number of concurrently analysed covariates, and precluded LASSO analysis of IgG titres in the absence of natural SARS-CoV-2 infection.

Nonetheless, this study highlights a role for IFN-I in the outcome of BNT162b2 +/− LAIV co-vaccination and identified sex differences in the primary IFN-I-producing cell type, pDCs. Free testosterone was seen to reduce pDC IFN-I, but DHT was associated with increased BNT162b2 antibody titres. As understanding of the molecular triggering events surrounding empirically developed vaccine adjuvant strategies increases, so will the need to better understand the complex role played by differential early IFN-I responses in determining the outcome of new vaccines.

## Materials and methods

### Ethics statement

Blood was sampled from adolescent participants with approval from the University of Oxford Medical Sciences Interdivisional Research Ethics Committee (reference R71346/RE001). Written informed parental consent with individual assent was obtained from each participant.

### Whole blood measurement of pDC IFN-I

pDC IFN-I was measured *via* whole blood stimulation as previously published (22). Briefly, blood was collected immediately following vaccination directly into heparin vacutainers (BD, Franklin Lakes, NJ, USA) containing R10 + Brefeldin A + CL097 and maintained at 37°C in a dry block heater during laboratory transport before further incubation up to 6 hours, red blood cell (RBC) lysis, and staining for flow cytometry *via* LSR II (flow cytometry antibodies are detailed in **Supplementary Table S3**).

### Determination of serum androgen concentrations

Blood was collected concurrently at the time of vaccination and a median of 37 days post-vaccination (“post-V1”) *via* SST II Advance tubes (BD). Serum was isolated *via* centrifugation of 2,000 *g* for 7 min and stored at −8°C. Steroids were extracted from 200 μL of serum and quantified using liquid chromatography–tandem mass spectrometry, as described previously (59, 60). Values below the lower limit of qualification (LLOQ) were set to half the LLOQ. The average of measurements for the day of vaccination and post-V1 was used for analysis.

The concentration of serum-free testosterone was calculated *via* the Vermeulen equation (34) using the concentration of serum sex hormone-binding globulin and albumin determined using a Cobas C system (Roche, Basel, Switzerland) *via* electrochemiluminescence (Elecsys SHBG) and bromocresol purple assay (ALBP), respectively. The average of measurements for the day of vaccination and post-V1 was used for analysis.

### Determination of anti-SARS-CoV-2 IgG responses and SARS-CoV-2 infection

Post-V1 antibody titres were taken from Jay et al. (32). Briefly, blood was collected *via* EDTA vacutainers (BD), and plasma was isolated by Ficoll (Lymphoprep) separation for determination of SARS-CoV-2 spike, receptor-binding domain (RBD), and nucleocapsid (N) *via* Meso Scale Discovery (MSD) V-plex immunoassay “Coronavirus Panel 3”, as described previously (32, 61).

SARS-CoV-2 infection was defined using the MSD cut-offs determined previously (61). Prior infection was defined as either anti-spike-IgG or anti-N-IgG above the cut-off, and infection post-V1 was defined as anti-N-IgG above the cut-off due to the inclusion of spike in BNT162b2.

### Data analysis and LASSO modelling

Flow cytometry data were analysed using FlowJo v10.7.1 (BD), and pDCs were defined as PBMC/Singlet/Live/CD56−/CD19−/CD3−/CD14−/CD11c−/CD123+/HLA-DR+, as previously mentioned (22). An example pDC gating strategy is given in **Supplementary Figure S1**. The fidelity of this gating strategy in identifying pDCs was confirmed by staining for BDCA2—which is increasingly used as a pDC-specific marker (62)—by pDCs identified *via* this strategy for samples from four healthy donors (male = 2 and female = 2) stimulated *via* the whole blood assay (see **Supplementary Figure S3**; **Supplementary Table S4**).

Graphs were plotted and analysed using R Studio running R v4.3.0 *via* base R (63) and package ggpubr. Two-group continuous variables were analysed *via* Student’s t-test (parametric data) and Wilcoxon test (non-parametric data). Where appropriate, Welch’s correction was applied for unequal variances, and Bonferoni’s correction was used to correct multiple comparisons.

The LASSO regression model assigns a penalty on the absolute value of each covariate’s regression coefficient and, hence, shrinks them towards zero, setting irrelevant variables to exactly zero. This way, variable selection is achieved, and any coefficients not regressed to zero, therefore, have an effect on the dependent variable in the model (35). The R package *glmnet* (36) was used to create relaxed-fit LASSO models of normalised data using *lambda.min* from model cross-validation.

## Data availability statement

The raw data supporting the conclusions of this article will be made available by the authors, without undue reservation.

## Ethics statement

The studies involving humans were approved by University of Oxford Medical Sciences Interdivisional Research Ethics Committee (reference R71346/RE001). The studies were conducted in accordance with the local legislation and institutional requirements. Written informed consent for participation in this study was provided by the participants’ legal guardians/next of kin.

## Author contributions

OS: Conceptualization, Data curation, Formal analysis, Investigation, Methodology, Visualization, Writing – original draft, Writing – review & editing. CJ: Investigation, Writing – review & editing. EA: Project administration, Writing – review & editing. AC: Investigation, Project administration, Writing – review & editing. NL: Investigation, Writing – review & editing. SE: Software, Writing – review & editing. LG: Investigation, Methodology, Writing – review & editing. AT: Investigation, Methodology, Writing – review & editing. SG: Investigation, Methodology, Writing – review & editing. SL: Investigation, Methodology, Writing – review & editing. LJ: Funding acquisition, Writing – review & editing. EB: Funding acquisition, Writing – review & editing. JF: Funding acquisition, Writing – review & editing. PK: Funding acquisition, Writing – review & editing. SD: Funding acquisition, Writing – review & editing. MC: Funding acquisition, Writing – review & editing. JH: Investigation, Methodology, Writing – review & editing. WA: Funding acquisition, Methodology, Project administration, Supervision, Writing – review & editing. AG: Software, Supervision, Writing – review & editing. PG: Conceptualization, Funding acquisition, Investigation, Project administration, Supervision, Writing – review & editing.
