# Supplementary material for: Gonadal androgens are associated with decreased type I interferon production by plasmacytoid dendritic cells and increased IgG titres to BNT162b2 following co-vaccination with live attenuated influenza vaccine in adolescents
Source: Front Immunol. 2024 Feb 28;15:1329805. doi: 10.3389/fimmu.2024.1329805 (PMC10933029; doi:10.3389/fimmu.2024.1329805)

***Supplementary Material***

**Supplementary Tables**

**Table S1 – LASSO regression co-efficient estimates for the effect of androgens on pDC IFN-I excluding free testosterone**

| **Covariate** | **Androgen Group** | **Coefficient** | **Interpretation** |
| --- | --- | --- | --- |
| Androstenedione | Gonadal | 0.00 | No effect |
| Testosterone | Gonadal | -0.32 | -0.32% IFN-α+ per nMol |
| Dihydrotestosterone (DHT) | Gonadal | 0.00 | No effect |
| Dehydroepiandrosterone (DHEA) | Adrenal | 0.00 | No effect |
| 11β-hydroxyandrostenedione  (11OHA4) | Adrenal | 0.00 | No effect |
| 11-ketoandrostenedione  (11KA4) | Adrenal | 0.00 | No effect |
| 11-ketotestosterone (11KT) | Adrenal | 0.00 | No effect |
| 11β-hydroxytestosterone (11OHT) | Adrenal | 0.00 | No effect |

**Table S2 – LASSO regression co-efficient estimates for the effect of androgens on post-V1 anti-Spike and anti-RBD IgG**

| **Covariate** | **Spike Coefficient** | **RBD Coefficient** |
| --- | --- | --- |
| Androstenedione | 0.00 | 0.00 |
| Testosterone | 0.00 | 0.00 |
| Free Testosterone | 0.00 | 0.00 |
| Dihydrotestosterone (DHT) | 7.42x10^-10 | 3.47x10^-10 |
| Dehydroepiandrosterone (DHEA) | 0.00 | 0.00 |
| 11β-hydroxyandrostenedione  (11OHA4) | 0.00 | 0.00 |
| 11-ketoandrostenedione  (11KA4) | 0.00 | 0.00 |
| 11-ketotestosterone (11KT) | 0.00 | 0.00 |
| 11β-hydroxytestosterone (11OHT) | 0.00 | 0.00 |

**Table S3 – Antibody clones used for pDC flow cytometry**

| **Marker** | **Fluorochrome** | **Clone** | **Supplier** | **Catalogue Number** |
| --- | --- | --- | --- | --- |
| Viability | NearIR | N/A | Thermo | L10119 |
| CD56 | Brilliant Violet 605 | HCD56 | BioLegend | 318334 |
| CD19 | APC | HIB19 | BioLegend | 302212 |
| CD3 | AlexaFluor 700 | OKT3 | BioLegend | 317340 |
| CD4 | Pe-Cy7 | OKT4 | BioLegend | 317414 |
| CD16 | Brilliant Violet 570 | 3G8 | BioLegend | 302036 |
| CD14 | Brilliant Violet 421 | MφP9 | BioLegend | 563743 |
| CD11c | Brilliant Violet 650 | B-Iy6 | BD | 563404 |
| HLA-DR | PE | L243 | BD | 307606 |
| CD123 | PE-Dazzle594 | 6H6 | BioLegend | 306034 |
| IFNa | FITC | REA1013 | BioLegend | 130-116-872 |
| TNFa | Brilliant Violet 711 | MAb11 | Miltenyi | 502940 |

**Table S4 – Percentage of pDCs gated by conventional strategy (as Supplementary Figure S1) also shown to express BDCA2 (as detailed in Supplementary Figure S3).**

| Donor | Sex | Conventionally-gated pDCs expressing BDCA2 (Rpt1) | Conventionally-gated pDCs expressing BDCA2 (Rpt2) | Average BDCA2 expression by conventionally-gated pDCs |
| --- | --- | --- | --- | --- |
| GI4207 | Male | 94.2% | 92.5% | 93.4% |
| GI4210 | Male | 94.1% | 93.2% | 93.7% |
| GI7891 | Female | 88.2% | 90.2% | 89.2% |
| GI7901 | Female | 93.7% | 95.1% | 94.4% |

**Supplementary Figures**

**Figure S1 – pDC Gating Strategy.** Representative gating strategy for isolating pDCs from lysed whole blood. After PBMC, Singlet, and Live Cell gating, pDCs were defined as: CD19-/CD56-/CD3-/CD14-/CD11c-/HLA-DR+/CD123+. Cytokine gates for IFNa and TNFa were set using unstimulated controls and applied to CL097-stimulated samples, as shown.


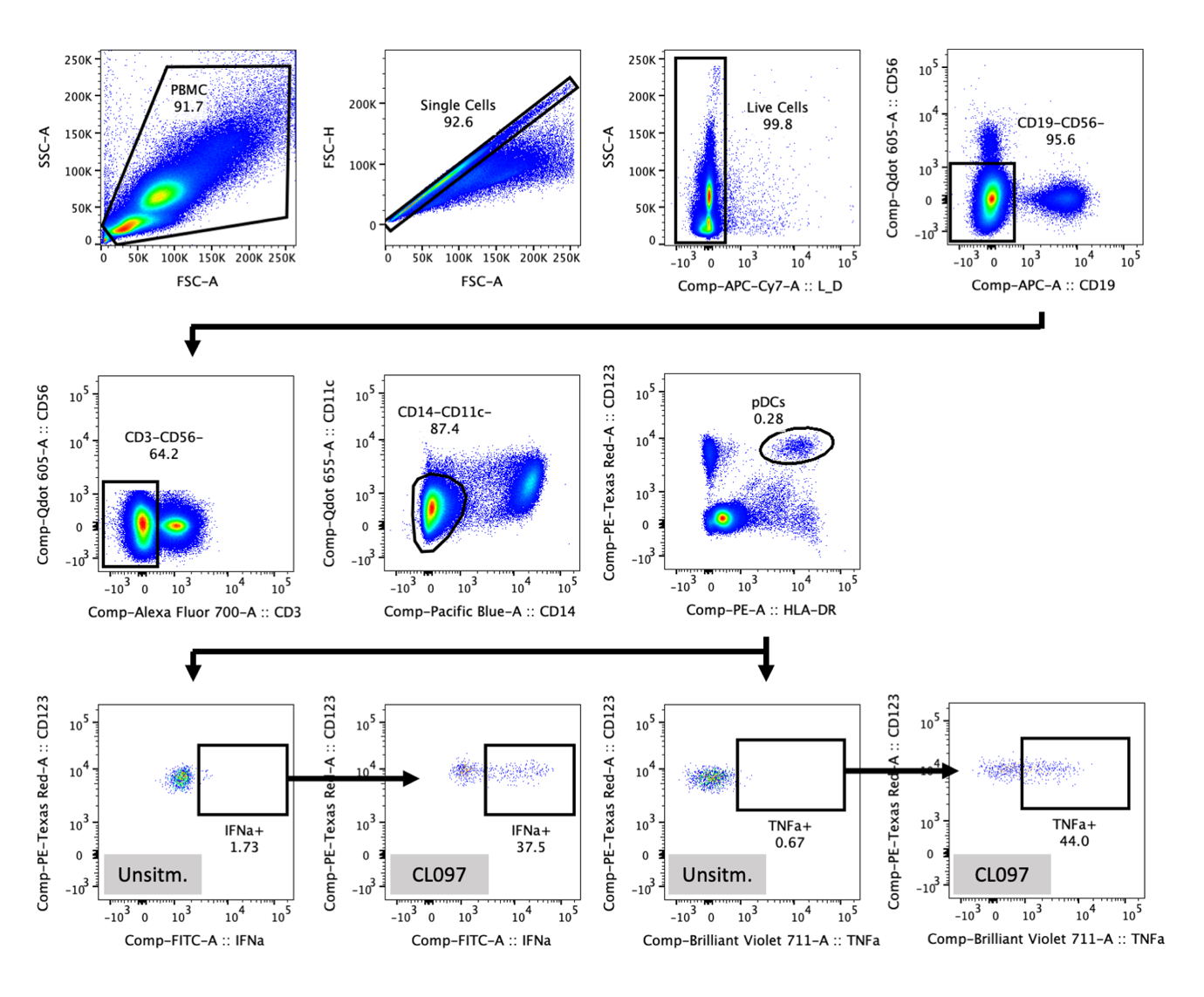


**Figure S2 – pDC phenotype of unvaccinated control individuals.** pDC flow cytometry data following whole blood TLR7 stimulation concurrently collected from two unvaccinated control individuals (male = 2) on the day of vaccination (D0) and the following day (D1). A) percentage of pDCs of live PBMC, B) MFI of pDC CD123, C) MFI of pDC HLA-DR, D) percentage of IFNa+ pDCs, E) MFI of pDC IFNa, F) percentage of pDC TNFa, G) MFI of pDC TNFa.


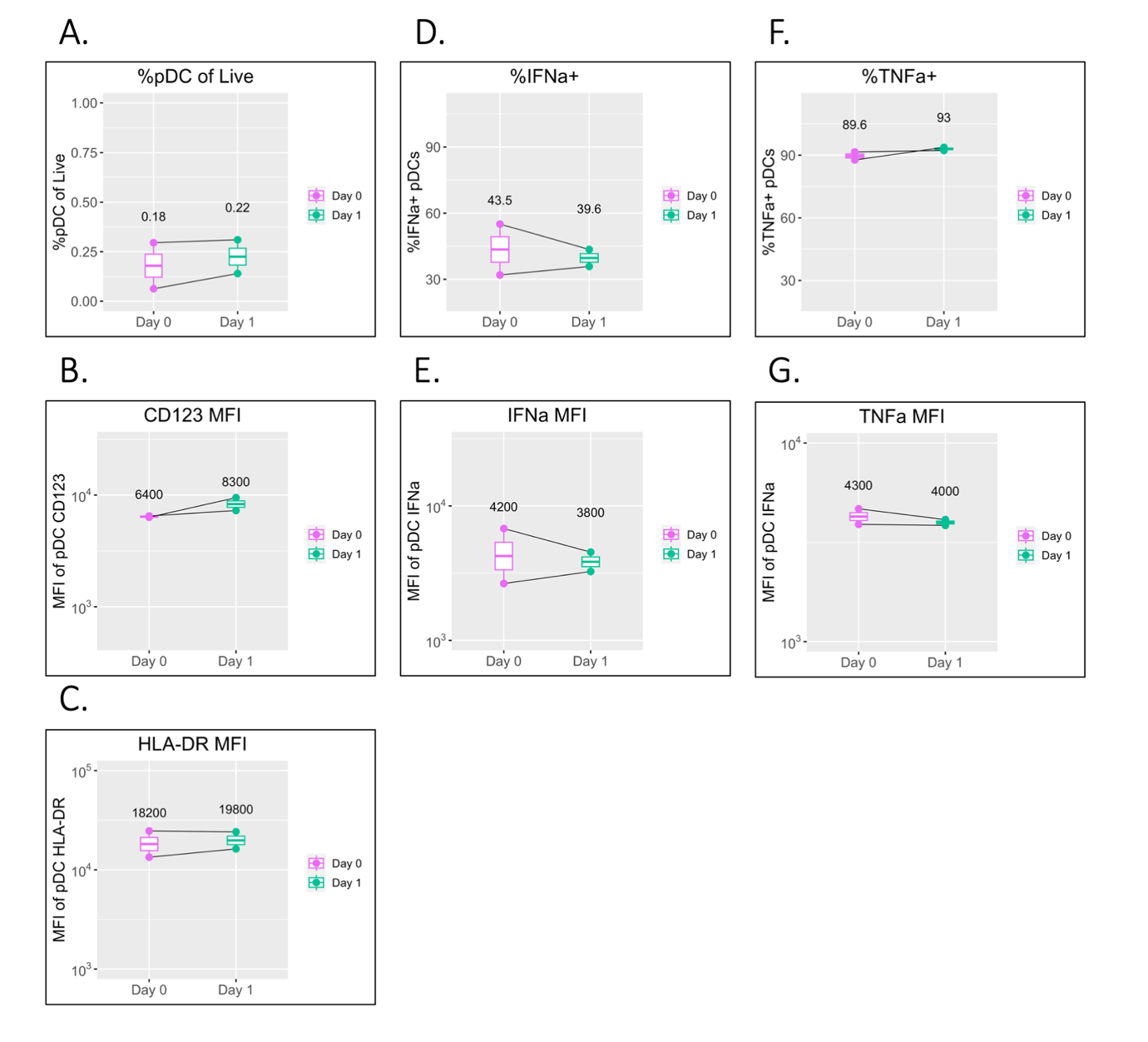


**Figure S3 – BDCA2 expression by pDCs gated with conventional strategy.** Blood from 4 healthy adult donors (male = 2 & female = 2) was TLR7 stimulated in duplicate via the whole blood assay and stained with a modified antibody panel to include BDCA2 (as Supplementary Table S3 except: i) CD14 moved to PE-Cy5 (BioLegend 301864) and ii) inclusion of BDCA2 Brilliant Violet 421 (BioLegend 354212). pDCs were then gated using conventional strategy (as Supplementary Figure S1) and BDCA2+ cells gated separately straight from live cells. Bottom Right panel: conventionally-gated pDCs (red) overlaid onto total live cells (blue) showing the BDCA2+ gate. BDCA2 expression by conventionally-gated pDCs was quantified for each replicate per donor and is detailed in Supplementary Table S4.


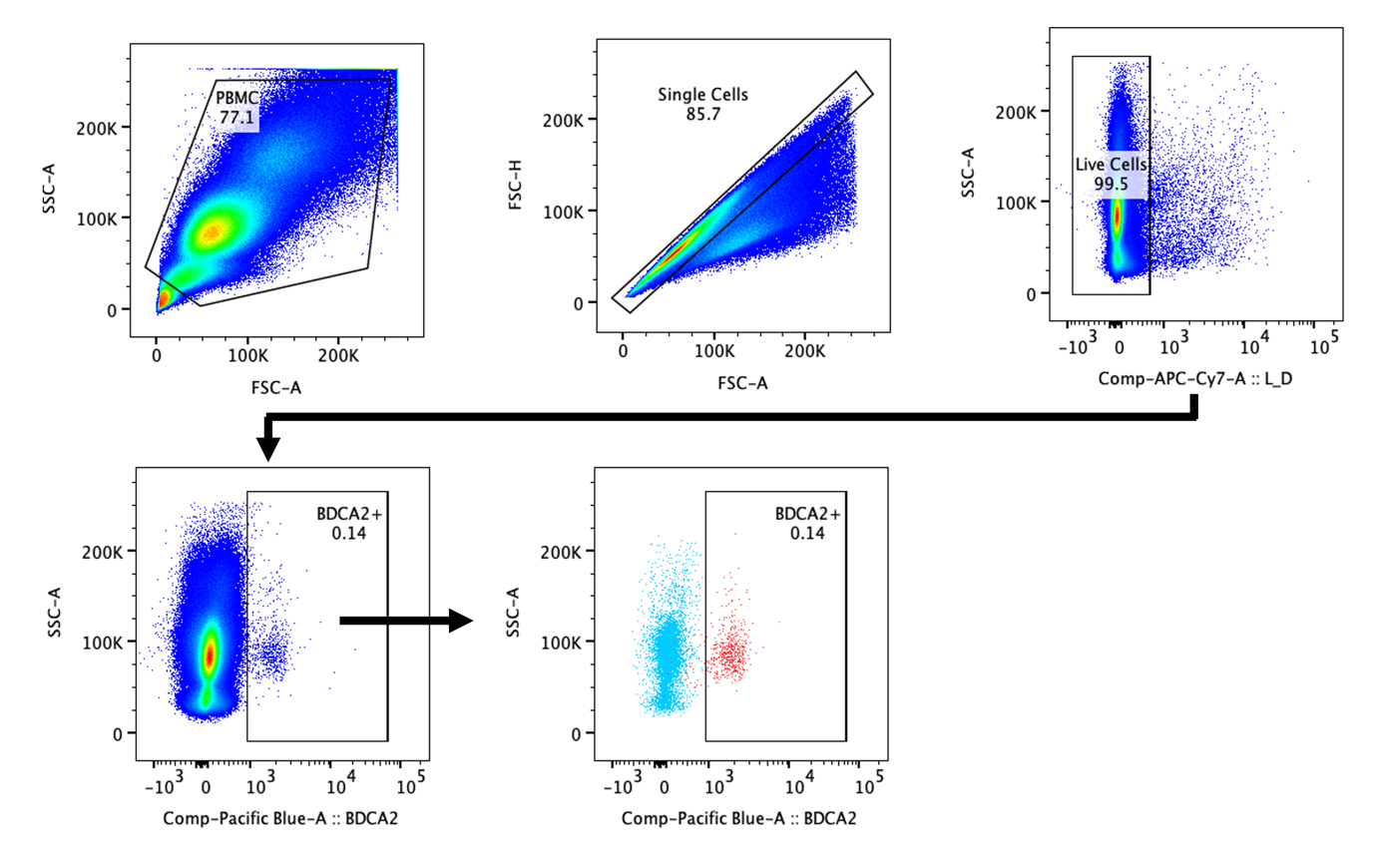

Supplement: Supplementary file 1 [file DataSheet_1.docx]
